# Are dietary inequalities among Australian adults changing? a nationally representative analysis of dietary change according to socioeconomic position between 1995 and 2011–13

**DOI:** 10.1186/s12966-018-0666-4

**Published:** 2018-04-02

**Authors:** Dana Lee Olstad, Rebecca M. Leech, Katherine M. Livingstone, Kylie Ball, Beth Thomas, Jane Potter, Xenia Cleanthous, Rachael Reynolds, Sarah A. McNaughton

**Affiliations:** 1 Department of Community Health Sciences, Cumming School of Medicine, University of Calgary, 3280 Hospital Drive NW, Calgary, AB T2N 4Z6 Canada; 2 Institute for Physical Activity and Nutrition (IPAN), School of Exercise and Nutrition Sciences, Deakin University, Geelong, Australia; 3 National Heart Foundation of Australia, Melbourne, Australia

**Keywords:** Australia, Adults, Dietary intake, Macronutrients, Energy, Fiber, Fruits and vegetables, Socioeconomic position, Nationally representative survey

## Abstract

**Background:**

Increasing inequalities in rates of obesity and chronic disease may be partly fuelled by increasing dietary inequalities, however very few nationally representative analyses of socioeconomic trends in dietary inequalities exist. The release of the 2011–13 Australian National Nutrition and Physical Activity Survey data allows investigation of change in dietary intake according to socioeconomic position (SEP) in Australia using a large, nationally representative sample, compared to the previous national survey in 1995. This study examined change in dietary intakes of energy, macronutrients, fiber, fruits and vegetables among Australian adults between 1995 and 2011–13, according to SEP.

**Methods:**

Cross-sectional data were obtained from the 1995 National Nutrition Survey, and the 2011–13 National Nutrition and Physical Activity Survey. Dietary intake data were collected via a 24-h dietary recall (*n* = 17,484 adults) and a dietary questionnaire (*n* = 15,287 adults). SEP was assessed according to educational level, equivalized household income, and area-level disadvantage. Survey-weighted linear and logistic regression models, adjusted for age, sex/gender and smoking status, examined change in dietary intakes over time.

**Results:**

Dietary intakes remained poor across the SEP spectrum in both surveys, as evidenced by high consumption of saturated fat and total sugars, and low fiber, fruit and vegetable intakes. There was consistent evidence (i.e. according to ≥2 SEP measures) of more favorable changes in dietary intakes of carbohydrate, polyunsaturated and monounsaturated fat in higher, relative to lower SEP groups, particularly in women. Intakes of energy, total fat, saturated fat and fruit differed over time according to a single SEP measure (i.e. educational level, household income, or area-level disadvantage). There were no changes in intake of total sugars, protein, fiber or vegetables according to any SEP measures.

**Conclusions:**

There were few changes in dietary intakes of energy, most macronutrients, fiber, fruits and vegetables in Australian adults between 1995 and 2011–13 according to SEP. For carbohydrate, polyunsaturated and monounsaturated fat, more favorable changes in intakes occurred in higher SEP groups. Despite the persistence of suboptimal dietary intakes, limited evidence of widening dietary inequalities is positive from a public health perspective.

**Trial registration:**

Clinical trials registration: ACTRN12617001045303.

**Electronic supplementary material:**

The online version of this article (10.1186/s12966-018-0666-4) contains supplementary material, which is available to authorized users.

## Background

Health inequalities are systematic differences in health among societal groups [1]. These differences in health are largely the consequence of social systems that differentially distribute material and social resources according to factors such as income, education, wealth and power, leading to social stratification [2]. In Australia [3, 4] and other developed nations [5–9], socioeconomically disadvantaged individuals have higher rates of obesity and chronic disease compared to their more advantaged counterparts. Although initiatives to reduce inequalities are underway worldwide [10], socioeconomic differences in obesity and health nevertheless persist and are widening in some cases [7, 11].

Increasing inequalities in rates of obesity and chronic disease may be partly fuelled by increasing dietary inequalities [12–14]. Examining socioeconomic trends in dietary intake might therefore reveal important targets for intervention that can guide public health policy. Recent analyses suggest that socioeconomic gradients in diet quality and in intakes of some foods and nutrients widened between 1999 and 2012 in the US [15–17]. For instance, Rehm et al. [15] found that although overall diet quality (American Heart Association diet score) improved between 1999 and 2012 in the US population, improvements were smaller for those with lower family incomes and lower educational attainment. Wang et al. [16] reported similarly (Alternate Healthy Eating Index Score), finding that the gap in diet quality between low and high socioeconomic status (a composite variable based on education and income level) adults in the US widened from 3.9 points in 1999 to 7.8 points in 2010. Comparable, recent, analyses have not been conducted in other nations, however. Such analyses are essential to understand how dietary inequalities have evolved over time within political, socioeconomic and dietary contexts that differ from those in the US, and can yield important insights with implications for policies intended to mitigate socioeconomic inequalities.

Dietary inequalities can be characterized at various levels, from the level of nutrients and moving upwards to consider foods, food groups, eating occasions, and dietary patterns. Quantifying change in inequalities at each of these levels is important, as associations between diet and health are underpinned by interactions among dietary components. The health impacts of dietary patterns, for instance, are mediated by their constituent foods, which are themselves comprised of macro and micronutrients with important physiological functions [18].

In Australia, the release of the 2011–13 National Nutrition and Physical Activity Survey (NNPAS) data have, for the first time, presented an opportunity to investigate change in dietary intake in adults according to socioeconomic position (SEP) using a large, nationally representative sample, compared to the previous national survey in 1995. These data provide a key opportunity to understand how dietary inequalities evolved over time within the Australian political, socioeconomic and dietary context. Therefore, the purpose of this study was to examine change in dietary inequalities in intakes of energy, macronutrients, fiber, fruits and vegetables among Australian adults between 1995 and 2011–13.

## Methods

### Survey design and sampling

Data for these analyses were provided by Australian adults (19–85 years) who participated in the 1995 National Nutrition Survey (NNS) [19], and adults who participated in the 2011–13 NNPAS [20]. These nationally representative, cross-sectional surveys were conducted using stratified multistage area-based sampling procedures of private dwellings in rural and urban locations in all Australian states and territories. The surveys were conducted under the authority of the Census and Statistics Act of 1905 and the procedures followed were in accordance with the Helsinki Declaration of 1975 as revised in 1983.

Participants in the 1995 NNS were a subsample of adults (*n* = 10,851; 61% response rate) who participated in the 1995 Australian National Health Survey. NNS participants completed an in-person 24-h dietary recall and provided information on a range of health behaviors and sociodemographic variables in a written questionnaire. Individuals who completed a dietary recall were also asked to complete a written dietary questionnaire with questions on usual fruit and vegetable intake, of whom 8332 did so (77% response rate). In 2011–13, 9341 adults participated in the NNPAS (77% response rate). Participants in the NNPAS completed a 24-h dietary recall, a brief dietary questionnaire with questions on usual fruit and vegetable intake, and provided information on a range of health behaviors and sociodemographic variables during in-person interviews.

Individuals were excluded if they were pregnant or breastfeeding, or were missing information on household income or neighborhood of residence. After excluding those who reported zero energy intake, the final sample for analysis of data from 24-h dietary recalls was 9277 in 1995 (4498 men and 4779 women), and 8207 in 2011–13 (3897 men and 4310 women) (Additional file 1). After excluding those who did not provide information on fruit and/or vegetable intakes, the final sample for analysis of change in fruit and vegetable intakes was 7078 individuals in 1995 (3346 men and 3732 women), and 8209 individuals in 2011–13 (3898 men and 4311 women) (Additional file 2).

### Dietary intake assessment

The current examination of dietary change spanned two levels of the nutritional hierarchy: nutrients and foods, as methodologic changes between surveys precluded analyses at other levels.

#### 24-h dietary recall

In 1995, dietary data were collected via an in-person three-stage multiple pass 24-h food recall with a trained dietitian (methods adapted from USDA [21]). In 2011–13, dietary data were collected via a computer-assisted in-person five-stage multiple pass 24-h food recall with trained interviewers from the Australian Bureau of Statistics (methods adapted from USDA [22]) The current study uses data from a single 24-h dietary recall, conducted on all days of the week, and during all seasons of the year.

Foods and beverages consumed were converted to nutrient intakes using databases prepared by the Australia New Zealand Food Authority (1995) [19] and Food Standards Australia New Zealand (2011–13) [23]. Intakes of energy (kJ) and of the following nutrients were considered: protein (% of energy (%E)), carbohydrate (%E), total sugars (%E), total fat (%E), saturated fat (SFA; %E), polyunsaturated fat (PUFA; %E), monounsaturated fat (MUFA; %E), and fiber (g/1000 kJ). In 1995, energy contributed by dietary fiber was not considered in the nutrient values provided by the Australian Bureau of Statistics. For comparability with the 2011–13 data, total energy intake, macronutrient intakes as a proportion of energy, and fiber density were re-calculated assuming that fiber contributed 8 kJ of energy per gram, which was the value used in 2011–13. Due to differences in the manner in which the contents of mixed dishes reported in 24-h recalls were disaggregated between surveys, data from 24-h recalls could not be used to examine change in intakes of particular foods or food groups over time.

#### Brief dietary questionnaire

In 1995, participants completed a self-administered, written dietary questionnaire in which they reported the number of daily serves of fruits and vegetables usually consumed in the past 12 months, with response options of: ≤ 1, 2–3, 4–5, ≥ 6 serves/d, or I don’t eat. These questions have been shown to provide valid estimates of fruit and vegetable intake [24, 25]. In 2011–13, participants reported the number of daily serves of fruits and vegetables they normally consumed to a trained interviewer, with response options of: 1, 2, 3, 4, 5, ≥ 6 serves/d, or I don’t eat. The 2011–13 questions were based on those used in 1995, and additionally specified that participants should include potatoes, but not fruit/vegetable juice, in their estimates. Usual daily fruit and vegetable consumption were dichotomized into ≥ 2 serves/d and ≥ 4 serves/d, respectively, as the response options in 1995 did not allow us to distinguish between those consuming 4 or 5 serves daily. These values were chosen to most closely resemble recommendations in the Australian Dietary Guidelines, which recommend consumption of at least 2 daily serves of fruit and at least 5 daily serves of vegetables [26].

### Socioeconomic position

We examined change according to three measures of SEP that have been associated with dietary intake in the literature [27–29], in previous analyses of the 1995 NNS [30–32] and 2011–13 NNPAS [33] and were comparable across the two surveys: educational level, household income, and area-level disadvantage.

#### Education

Educational level in both surveys was based on whether participants had completed secondary school (yes, no) and their highest post-school qualification (1995: 29 pre-coded response options across three questions; 2011–13: open-ended responses). Responses were categorized as low (less than secondary), medium (secondary/trade/diploma), or high (tertiary).

#### Income

Individuals reported total weekly gross household income in both surveys. Differences in household types and compositions, and their requirements relative to income, were taken into account by calculating household income equivalence scales [34], which were expressed as deciles of gross equivalized household income. Deciles were subsequently combined into three income groups categorized as low (decile 1–3), medium (decile 4–7), or high (decile 8–10).

#### Area-level disadvantage

The Australian Bureau of Statistics’ Socio-Economic Index for Areas (SEIFA) classifies areas according to relative levels of socioeconomic deprivation using variables collected in the Australian census [35]. The SEIFA Index of Relative Socio-Economic Disadvantage is divided into quintiles ordered from most (quintile 1) to least disadvantaged (quintile 5). Higher quintiles are indicative of areas with lower levels of disadvantage, such as where fewer individuals have low incomes, low educational attainment, or work in unskilled occupations. Individuals were assigned to quintiles based on their area of residence in both surveys.

### Potential confounders

Age (continuous) and smoking status (current, former, never) were considered as potential confounders. Sex/gender was included as a potential confounder in overall analyses, and was used as a stratifying variable in sex/gender-specific analyses. Although the survey asked participants to report sex using a single question with binary response categories (male or female), the biological factor of sex cannot be analytically disentangled from the social construct of gender. This is particularly the case for the current analyses, as dietary intake and vulnerability to socioeconomic disadvantage are functions of both biological (sex) and behavioral (gender) attributes. Our analyses therefore capture the joint effects of sex and gender, but our interpretations focus on the effects of gendered social roles and behaviors, rather than biological differences associated with sex.

#### Energy intake underreporting

The probability of underreporting, defined as a ratio of reported energy intake to basal metabolic rate of < 1.2 [36–39], did not differ over time according to SEP in our data set (*n* = 16,390, as 1094 participants were missing BMI which is required to estimate basal metabolic rate). Therefore, given that our aim was to estimate change in dietary inequalities over time rather than absolute intakes at a particular point in time, and in keeping with previous analyses [15], no adjustment was made for energy intake misreporting. This also allowed us to retain participants with missing BMI data in the analysis, thereby reducing possible selection bias.

### Statistical analyses

Descriptive statistics for nutrient intakes are presented as weighted means and proportions with 95% CI. Linear regression models (Wald test of association) examined change in dietary intakes of energy, protein, carbohydrate, total sugars, fat, fat subtypes and fiber (continuous outcomes) according to three categorical measures of SEP exposures (education level, household income, area-level disadvantage) between 1995 and 2011–13 (*n* = 17,484). Logistic regression (Wald test of association) examined change in the probability of meeting fruit intake recommendations (≥ 2 serves/d), or consuming ≥ 4 serves/d of vegetables between 1995 and 2011–13 (*n* = 15,287), also according to the three categorical measures of SEP. Person-specific weights, adjusted for probability of selection and non-response, were used to ensure estimates were representative of the Australian population. Overall models adjusted for age (continuous), sex/gender (binary), and smoking (categorical). Sex/gender-stratified models were adjusted for age and smoking. To assess whether the association of SEP with dietary intake differed over time, all models included a categorical interaction term for SEP by time. If the results of the overall F test were statistically significant at the *p* < 0.05 level, pairwise comparisons were performed to examine change in intakes over time according to SEP level.

Although a large number of tests were conducted, they were all pre-planned based on prior evidence of associations [15, 27]. Adjustments for multiple comparisons can reduce the likelihood of Type 1 errors, however they do so at the expense of increasing the likelihood of Type 2 errors [40]. Given that our data represent real dietary intake data, many of which have been associated with SEP in previous studies [15, 27], it would be wrong to assume that Type 1 errors were of greater concern than Type 2 errors [40]. Therefore in keeping with similar studies [15], data were not adjusted for multiple comparisons. Results were, however, interpreted cautiously considering *p*-values, theoretical plausibility and effect sizes. Moreover, we focussed our discussion on changes in dietary intakes for which evidence of change was strongest (i.e. changes observed for multiple SEP measures), and avoided discussing non-significant trends. Survey-weighted, adjusted means and 95% CI are presented, using the post-hoc margins command in Stata. *P* < 0.05 was considered a statistically significant finding for all analyses. Analyses were conducted in Stata (version 13.0; Stata Corp, TX, USA).

## Results

### Descriptive characteristics

Survey-weighted sociodemographic characteristics and dietary intakes of participants in 1995 and 2011–13 are presented in Table 1. Both samples were nearly evenly distributed according to sex/gender, however the mean age in 2011–13 was higher at 47.3 years (95% CI: 46.8, 47.8), compared to 44.3 years (95% CI: 43.8, 44.8) in 1995. Educational level increased over time, while smoking declined. Reported energy intake was lower in 2011–13 (8737 kJ/d, 95% CI: 8629–8844 KJ/d) compared to 1995 (9444 kJ/d, 95% CI: 9338–9550 kJ/d).

**Table 1:** Participant characteristics and dietary intake in the 1995 National Nutrition Survey and the 2011–13 National Nutrition and Physical Activity Survey

Variables	1995 National Nutrition Survey			2011–13 National Nutrition and Physical Activity Survey		
	Men	Women	Overall	Men	Women	Overall
	*n* = 4498	*n* = 4779	*n* = 9277	*n* = 3897	*n* = 4310	*n* = 8207
Sex/gender^a^	4498 (50.9) [49.8, 51.9]	4779 (49.2) [48.1, 50.2]		3897 (51.7) [50.2, 53.1]	4310 (48.3) [46.9, 49.8]	
Age^b^	43.2 [42.6, 43.8]	45.4 [44.8, 46.0]	44.3 [43.8, 44.8]	46.7 [46.0, 47.4]	47.9 [47.2, 48.6]	47.3 [46.8, 47.8]
Equivalized weekly household income^a^						
Low (decile 1–3)	1115 (24.9) [23.4, 26.5]	1492 (31.5) [29.9, 33.1]	2607 (28.1) [26.9, 29.4]	1014 (24.5) [22.8, 26.3]	1520 (31.5) [29.7, 33.3]	2534 (27.9) [26.6, 29.2]
Medium (decile 4–7)	1731 (38.2) [36.5, 40.0]	1793 (37.7) [36.1, 39.4]	3524 (38.0) [36.6, 39.3]	1484 (41.1) [39.1, 43.2]	1626 (40.0) [38.0, 42.0]	3110 (40.6) [39.2, 42.0]
High (decile 8–10)	1652 (36.9) [35.2, 38.6]	1494 (30.9) [29.3, 32.4]	3146 (33.9) [32.6, 35.3]	1399 (34.3) [32.4, 36.4]	1164 (28.5) [26.7, 30.4]	2563 (31.5) [30.2, 32.9]
Educational level^a^						
Low (less than secondary)	2172 (45.9) [44.2, 47.7]	2703 (55.0) [53.2, 56.7]	4875 (50.4) [49.1, 51.7]	936 (22.4) [20.7, 24.1]	1359 (29.0) [27.3, 30.8]	2295 (25.6) [24.4, 26.9]
Medium (secondary/diploma/trade)	2001 (46.1) [44.3, 47.9]	1784 (39.4) [37.7, 41.1]	3785 (42.8) [41.5, 44.1]	2017 (54.1) [52.0, 56.2]	1774 (43.3) [41.3, 45.4]	3791 (48.9) [47.4, 50.4]
High (tertiary)	325 (8.0) [7.0, 9.1]	292 (5.7) [5.0, 6.5]	617 (6.8) [6.2, 7.6]	944 (23.5) [21.8, 25.3]	1177 (27.7) [25.9, 29.5]	2121 (25.5) [24.3, 26.8]
Area-level disadvantage^a^						
1st quintile (most disadvantaged)	829 (17.7) [16.3, 19.1]	949 (19.3) [18.0, 20.7]	1778 (18.5) [17.4, 19.6]	717 (18.3) [16.7, 20.0]	865 (19.1) [17.5, 20.7]	1582 (18.7) [17.6, 19.8]
2nd quintile	863 (18.8) [17.5, 20.3]	924 (19.3) [17.9, 20.7]	1787 (19.1) [17.9, 20.3]	819 (21.0) [19.4, 22.8]	900 (20.0) [18.4, 21.6]	1719 (20.5) [19.4, 21.7]
3rd quintile	881 (17.8) [16.4, 19.2]	929 (18.1) [16.8, 19.5]	1810 (18.0) [16.9, 19.1]	779 (20.7) [19.1, 22.5]	854 (21.0) [19.4, 22.7]	1633 (20.9) [19.7, 22.1]
4th quintile	943 (21.4) [19.9, 23.0]	1010 (21.0) [19.7, 22.5]	1953 (21.2) [20.0, 22.5]	719 (19.6) [18.0, 21.4]	734 (18.2) [16.6, 19.8]	1453 (18.9) [17.8, 20.1]
5th quintile (least disadvantaged)	982 (24.3) [22.7, 26.1]	967 (22.2) [20.7, 23.8]	1949 (23.3) [22.0, 24.7]	863 (20.3) [18.7, 22.1]	957 (21.8) [20.2, 23.6]	1820 (21.1) [19.9, 22.3]
Smoking^a^						
Never	1710 (39.8) [38.1, 41.6]	2627 (56.3) [54.6, 58.0]	4337 (47.9) [46.6, 49.2]	1570 (43.8) [41.7, 45.9]	2342 (57.1) [55.1, 59.1]	3912 (50.2) [48.8, 51.7]
Former	1564 (31.7) [30.1, 33.3]	1068 (20.9) [19.6, 22.2]	2632 (26.4) [25.3, 27.5]	1489 (36.3) [34.3, 38.3]	1227 (27.4) [25.7, 29.2]	2716 (32.0) [30.7, 33.4]
Current	1224 (28.5) [26.8, 30.2]	1084 (22.8) [21.4, 24.3]	2308 (25.7) [24.5, 26.9]	838 (19.9) [18.3, 21.6]	741 (15.4) [14.1, 16.9]	1579 (17.8) [16.7, 18.9]
Energy (kJ)^b^	11,272 [11,118, 11,427]	7553 [7452, 7653]	9444 [9338, 9550]	9988 [9830, 10,147]	7398 [7281, 7516]	8737 [8629, 8844]
Carbohydrate (%E)^b,c^	44.4 [44.0, 44.7]	46.1 [45.8, 46.4]	45.2 [45.0, 45.5]	43.3 [42.8, 43.7]	43.5 [43.0, 43.9]	43.4 [43.1, 43.7]
Total sugars (%E)^b,c^	19.1 [18.8, 19.4]	20.4 [20.1, 20.7]	19.8 [19.5, 20.0]	18.6 [18.2, 18.9]	19.8 [19.4, 20.2]	19.2 [18.9, 19.4]
Protein (%E)^b,c^	16.7 [16.5, 16.8]	16.8 [16.6, 17.0]	16.7 [16.6, 16.9]	18.2 [17.9, 18.4]	18.5 [18.3, 18.8]	18.4 [18.2, 18.5]
Fat (%E)^b,c^	31.8 [31.5, 32.1]	31.7 [31.4, 32.0]	31.8 [31.5, 32.0]	30.5 [30.1, 30.9]	31.2 [30.8, 31.5]	30.8 [30.6, 31.1]
SFA (%E)^b,c^	12.5 [12.4, 12.7]	12.4 [12.2, 12.5]	12.4 [12.3, 12.6]	11.5 [11.3, 11.7]	11.5 [11.3, 11.7]	11.5 [11.4, 11.6]
MUFA (%E)^b,c^	11.7 [11.6, 11.8]	11.4 [11.3, 11.5]	11.5 [11.4, 11.6]	11.7 [11.5, 11.9]	11.9 [11.7, 12.0]	11.8 [11.7, 11.9]
PUFA (%E)^b,c^	4.8 [4.7, 4.8]	4.9 [4.9, 5.0]	4.9 [4.8, 4.9]	4.7 [4.6, 4.8]	5.0 [4.9, 5.1]	4.8 [4.7, 4.9]
Fiber density (g/1000 kJ)^b^	1.91 [1.88, 1.94]	2.25 [2.22, 2.28]	2.08 [2.05, 2.10]	2.62 [2.57, 2.67]	2.95 [2.89, 3.01]	2.78 [2.74, 2.82]
	*n* = 3346	*n* = 3732	*n* = 7078	*n* = 3898	*n* = 4311	*n* = 8209
Fruit (≥ 2 serves/d)^a^	1578 (45.9) [43.8, 48.0]	2013 (54.6) [52.6, 56.6]	3591 (50.2) [48.7, 51.7]	1641 (42.3) [40.3, 44.4]	2331 (54.2) [52.2, 56.2]	3972 (48.1) [46.6, 49.5]
Vegetables (≥ 4 serves/d)^a^	572 (15.4) [14.0, 16.9]	823 (21.9) [20.3, 23.6]	1395 (18.6) [17.5, 19.8]	627 (16.5) [15.0, 18.1]	956 (21.6) [20.0, 23.3]	1583 (19.0) [17.8, 20.1]

^a^Values represent n, weighted proportions and 95% CI, n (%) [95% CI]

^b^Values represent weighted means and 95% CI, mean [95% CI]

^c^%E: proportion of energy intake

### Change in dietary intakes according to socioeconomic position between 1995 and 2011–13

#### Educational level

Dietary intakes of energy, carbohydrate, MUFA, and PUFA varied over time by educational level (Table 2). Energy intake among individuals with a low level of education declined by 419 kJ/d between 1995 and 2011–13 (*p* < 0.001), whereas the reduction among those with a middle and high educational level was more than double at 880 kJ/d and 870 kJ/d, respectively (p < 0.001). The proportion of energy from carbohydrate did not change among those with a low level of education, compared to declines of 2.2%E and 2.1%E among medium and high education groups (p < 0.001), respectively. Intake of MUFA did not change over time in the low or high education groups, whereas it increased by 0.4%E in the middle education group (*p* = 0.001). Conversely, PUFA intakes declined by 0.4%E in the low education group (*p* < 0.001), but remained unchanged in the middle and high education groups. There were no changes over time according to educational level in dietary intakes of total sugars, protein, total fat, SFA, fiber, fruit or vegetables.

**Table 2:** Dietary intake according to level of education in Australian adults in the 1995 National Nutrition Survey and the 2011–13 National Nutrition and Physical Activity Survey^a^

	OVERALL, mean [95% CI]						
	Low education		Medium education		High education		
	1995 *n* = 4875	2011–13 *n* = 2295	1995 *n* = 3785	2011–13 *n* = 3791	1995 *n* = 617	2011–13 *n* = 2121	p interaction SEP*Time
Energy (kJ)	9195 [9063, 9327]	8776 [8579, 8974]***	9549 [9397, 9700]	8669 [8525, 8813]***	9861 [9488, 10,235]	8991 [8790, 9193]***	**0.007**
Carbohydrate (%E)	45.1 [44.8, 45.5]	44.5 [43.9, 45.2]	45.4 [45.0, 45.8]	43.2 [42.7, 43.7]***	44.6 [43.6, 45.6]	42.5 [41.9, 43.2]***	**0.001**
Total sugars (%E)	19.9 [19.5, 20.2]	20.0 [19.4, 20.5]	19.7 [19.4, 20.1]	19.2 [18.9, 19.6]	18.8 [17.9, 19.7]	18.3 [17.8, 18.8]	0.34
Protein (%E)	16.9 [16.8, 17.1]	18.3 [17.9, 18.7]	16.7 [16.5, 16.8]	18.4 [18.1, 18.6]	16.3 [15.9, 16.7]	18.3 [17.9, 18.6]	0.17
Fat (%E)	32.1 [31.8, 32.4]	30.8 [30.3, 31.4]	31.2 [30.9, 31.5]	30.7 [30.3, 31.1]	32.0 [31.2, 32.8]	31.3 [30.8, 31.8]	0.19
SFA (%E)	12.5 [12.4, 12.7]	11.9 [11.7, 12.2]	12.2 [12.0, 12.4]	11.4 [11.2, 11.6]	12.4 [12.0, 12.8]	11.4 [11.1, 11.6]	0.35
MUFA (%E)	11.7 [11.5, 11.8]	11.6 [11.4, 11.9]	11.3 [11.2, 11.5]	11.7 [11.5, 11.9]**	11.7 [11.3, 12.1]	12.1 [11.9, 12.3]	**0.036**
PUFA (%E)	4.9 [4.8, 5.0]	4.5 [4.4, 4.7]***	4.8 [4.7, 4.9]	4.8 [4.7, 4.9]	4.9 [4.7, 5.1]	5.0 [4.9, 5.2]	**0.0001**
Fiber density (g/1000 kJ)	2.1 [2.0, 2.1]	2.6 [2.6, 2.7]	2.1 [2.1, 2.2]	2.8 [2.7, 2.8]	2.2 [2.1, 2.3]	2.9 [2.8, 3.0]	0.22
	Probability [95% CI]						
	*n* = 3718	n = 2295	*n* = 2877	*n* = 3792	*n* = 483	*n* = 2122	p interaction SEP*Time
Fruit (≥ 2 serves/d)	0.49 [0.47, 0.51]	0.44 [0.41, 0.47]	0.52 [0.50, 0.55]	0.46 [0.44, 0.48]	0.57 [0.52, 0.63]	0.53 [0.50, 0.56]	0.68
Vegetables (≥ 4 serves/d)	0.20 [0.19, 0.22]	0.18 [0.15, 0.20]	0.18 [0.16, 0.20]	0.19 [0.17, 0.21]	0.16 [0.12, 0.20]	0.19 [0.17, 0.22]	0.05
	MEN, mean [95% CI]						
	Low education		Medium education		High education		
	1995 *n* = 2172	2011–13 *n* = 936	1995 *n* = 2001	2011–13 *n* = 2017	1995 *n* = 325	2011–13 *n* = 944	p interaction SEP*Time
Energy (kJ)	10,995 [10,781, 11,209]	10,242 [9915, 10,568]	11,240 [11,020, 11,460]	9981 [9771, 10,191]	11,525 [10,961, 12,089]	10,174 [9844, 10,504]	0.09
Carbohydrate (%E)	44.2 [43.7, 44.7]	44.2 [43.3, 45.1]	44.7 [44.2, 45.2]	43.1 [42.5, 43.7]	43.8 [42.5, 45.1]	42.7 [41.8, 43.6]	0.06
Total sugars (%E)	19.5 [19.0, 20.0]	19.3 [18.5, 20.1]	18.9 [18.5, 19.4]	18.8 [18.3, 19.3]	17.8 [16.7, 18.9]	17.4 [16.7, 18.1]	0.97
Protein (%E)	16.8 [16.6, 17.1]	18.2 [17.6, 18.7]	16.6 [16.4, 16.9]	18.1 [17.8, 18.5]	16.4 [15.8, 16.9]	18.2 [17.7, 18.7]	0.58
Fat (%E)	32.1 [31.7, 32.5]	30.8 [30.1, 31.6]	31.3 [30.9, 31.7]	30.3 [29.8, 30.8]	32.1 [31.1, 33.1]	30.8 [30.1, 31.6]	0.88
SFA (%E)	12.6 [12.4, 12.8]	12.0 [11.6, 12.4]	12.4 [12.1, 12.6]	11.4 [11.2, 11.7]	12.4 [11.8, 13.0]	11.2 [10.9, 11.5]	0.22
MUFA (%E)	11.8 [11.6, 12.0]	11.6 [11.3, 12.0]	11.5 [11.3, 11.6]	11.6 [11.4, 11.8]	11.8 [11.4, 12.3]	12.1 [11.7, 12.4]	0.36
PUFA (%E)	4.9 [4.7, 5.0]	4.5 [4.3, 4.7]	4.6 [4.5, 4.7]	4.6 [4.5, 4.8]	5.0 [4.7, 5.3]	4.9 [4.7, 5.1]	0.05
Fiber density (g/1000 kJ)	1.9 [1.9, 2.0]	2.4 [2.4, 2.5]	2.0 [1.9, 2.0]	2.6 [2.5, 2.7]	2.0 [2.0, 2.1]	2.7 [2.6, 2.8]	0.09
	Probability [95% CI]						
	*n* = 1622	n = 936	*n* = 1485	n = 2017	*n* = 239	*n* = 945	p interaction SEP*Time
Fruit (≥ 2 serves/d)	0.45 [0.42, 0.48]	0.38 [0.34, 0.42]	0.48 [0.45, 0.51]	0.40 [0.37, 0.43]	0.53 [0.45, 0.60]	0.48 [0.43, 0.52]	0.83
Vegetables (≥ 4 serves/d)	0.18 [0.16, 0.20]	0.16 [0.13, 0.19]	0.14 [0.12, 0.16]	0.16 [0.14, 0.18]	0.14 [0.09, 0.20]	0.16 [0.13, 0.19]	0.26
	WOMEN, mean [95% CI]						
	Low education		Medium education		High education		
	1995 *n* = 2703	2011–13 *n* = 1359	1995 *n* = 1784	2011–13 *n* = 1774	1995 *n* = 292	2011–13 *n* = 1177	p interaction SEP*Time
Energy (kJ)	7278 [7151, 7404]	7080 [6867, 7292]	7801 [7617, 7985]	7421 [7239, 7602]	8115 [7778, 8452]	7760 [7527, 7994]	0.55
Carbohydrate (%E)	46.0 [45.6, 46.4]	45.0 [44.1, 45.9]*	46.2 [45.7, 46.8]	43.2 [42.5, 43.9]***	45.6 [44.2, 47.1]	42.4 [41.6, 43.3]***	**0.004**
Total sugars (%E)	20.3 [18.9, 20.7]	20.6 [19.8, 21.4]	20.6 [20.1, 21.1]	19.7 [19.1, 20.2]	20.1 [18.7, 21.4]	19.3 [18.6, 19.9]	0.09
Protein (%E)	17.0 [16.8, 17.2]	18.4 [17.9, 18.9]	16.7 [16.4, 17.0]	18.7 [18.3, 19.1]	16.1 [15.6, 16.7]	18.4 [17.9, 18.8]	0.10
Fat (%E)	32.1 [31.7, 32.5]	30.9 [30.1, 31.6]	31.1 [30.6, 31.6]	31.1 [30.5, 31.7]	31.7 [30.5, 32.9]	31.7 [31.1, 32.4]	0.05
SFA (%E)	12.5 [12.3, 12.7]	11.8 [11.5, 12.2]	12.0 [11.8, 12.3]	11.4 [11.1, 11.7]	12.4 [11.8, 13.0]	11.5 [11.2, 11.9]	0.88
MUFA (%E)	11.6 [11.4, 11.7]	11.6 [11.3, 12.0]	11.1 [10.9, 11.3]	11.9 [11.6, 12.1]***	11.5 [10.9, 12.0]	12.1 [11.8, 12.5]*	**0.031**
PUFA (%E)	5.0 [4.9, 5.1]	4.6 [4.4, 4.8]**	4.9 [4.8, 5.1]	5.1 [4.9, 5.3]	4.8 [4.5, 5.1]	5.2 [5.0, 5.4]*	**0.0001**
Fiber density (g/1000 kJ)	2.2 [2.2, 2.3]	2.8 [2.7, 2.9]	2.3 [2.3, 2.4]	2.9 [2.8, 3.0]	2.4 [2.3, 2.5]	3.0 [2.9, 3.2]	0.87
	Probability [95%CI]						
	*n* = 2096	n = 1359	*n* = 1392	*n* = 1775	*n* = 244	n = 1177	p interaction SEP*Time
Fruit (≥ 2 serves/d)	0.53 [0.51, 0.56]	0.51 [0.47, 0.54]	0.57 [0.54, 0.60]	0.52 [0.49, 0.55]	0.62 [0.55, 0.69]	0.59 [0.55, 0.62]	0.78
Vegetables (≥ 4 serves/d)	0.23 [0.20, 0.25]	0.19 [0.17, 0.22]	0.22 [0.19, 0.25]	0.22 [0.19, 0.24]	0.18 [0.12, 0.23]	0.23 [0.20, 0.26]	0.09

^a^*Abbreviations: %E* % of energy, *SEP* socioeconomic position

Asterisks indicate significantly different compared to 1995 value *p < 0.05, **p ≤ 0.01, ***p < 0.001

Values represent survey-weighted, covariate adjusted means (or probabilities where indicated) and 95% CI

Linear and logistic regression models were adjusted for age, sex/gender (except in stratified analyses), and smoking. Where results of the overall F test were statistically significant, pairwise comparisons were performed to examine change in intakes over time according to educational level

Low education: less than secondary; Medium education: secondary/diploma/trade; High education: tertiary

Bold data indicates statistically significant differences (*p*  < 0.05)

In stratified analyses, dietary intake did not vary by educational level over time amongst men, however consumption of carbohydrate, MUFA and PUFA varied over time by education level among women (Table 2).

#### Household income

Trends in consumption of carbohydrate, SFA, PUFA and fruit varied over time by household income level (Table 3). Carbohydrate intake as a proportion of energy declined by 0.9% between 1995 and 2011–13 among those with low incomes (*p* = 0.02), whereas those with medium and high incomes showed declines of 1.7%E and 2.9%E (*p* < 0.001), respectively. Intake of SFA was unchanged in the low income group, while SFA intakes declined by 1.1%E among those with medium and high incomes over time (p < 0.001). PUFA intake declined by 0.3%E in those with low incomes (*p* < 0.001), remained stable in the medium income group, and increased by 0.2%E among those with a high income (p = 0.02). The probability of meeting fruit intake recommendations was unchanged in the low income group, but decreased over time in the medium and high income groups by 0.07 (*p* < 0.001) and 0.05 (p = 0.02), respectively. There were no changes over time according to income level in dietary intakes of energy, total sugars, protein, total fat, MUFA, fiber or vegetables.

**Table 3:** Dietary intake according to equivalized household income in Australian adults in the 1995 National Nutrition Survey and the 2011–13 National Nutrition and Physical Activity Survey^a^

	OVERALL, mean [95% CI]						
	Low income		Medium income		High income		
	1995 *n* = 2607	2011–13 *n* = 2534	1995 *n* = 3524	2011–13 *n* = 3110	1995 *n* = 3146	2011–13 *n* = 2563	p interaction SEP*Time
Energy (kJ)	9025 [8846, 9204]	8564 [8377, 8751]	9395 [9241, 9550]	8756 [8598, 8915]	9695 [9531, 9860]	8995 [8813, 9178]	0.36
Carbohydrate (%E)	46.1 [45.6, 46.6]	45.2 [44.6, 45.8]*	45.5 [45.1, 45.9]	43.8 [43.3, 44.2]***	44.2 [43.8, 44.6]	41.3 [40.7, 41.9]***	**0.001**
Total sugars (%E)	19.9 [19.4, 20.4]	19.9 [19.3, 20.4]	19.9 [19.6, 20.3]	19.3 [18.9, 19.7]	19.4 [19.0, 19.7]	18.5 [18.0, 18.9]	0.16
Protein (%E)	16.6 [16.4, 16.9]	17.9 [17.6, 18.2]	16.7 [16.5, 16.9]	18.3 [18.0, 18.5]	17.0 [16.7, 17.2]	18.8 [18.4, 19.1]	0.19
Fat (%E)	31.5 [31.1, 31.9]	30.8 [30.3, 31.3]	31.9 [31.6, 32.3]	30.7 [30.3, 31.2]	31.7 [31.3, 32.0]	31.1 [30.6, 31.6]	0.30
SFA (%E)	12.2 [11.9, 12.4]	11.8 [11.6, 12.1]	12.6 [12.4, 12.8]	11.5 [11.3, 11.7]***	12.4 [12.2, 12.6]	11.3 [11.1, 11.6]***	**0.001**
MUFA (%E)	11.5 [11.3, 11.7]	11.6 [11.4, 11.9]	11.6 [11.4, 11.7]	11.7 [11.5, 11.9]	11.5 [11.4, 11.7]	12.0 [11.8, 12.2]	0.19
PUFA (%E)	4.9 [4.8, 5.0]	4.6 [4.4, 4.7]***	4.9 [4.8, 5.0]	4.8 [4.7, 4.9]	4.8 [4.7, 4.9]	5.0 [4.9, 5.2]*	**0.0001**
Fiber density (g/1000 kJ)	2.1 [2.1, 2.2]	2.8 [2.7, 2.9]	2.1 [2.0, 2.1]	2.7 [2.7, 2.8]	2.1 [2.1, 2.2]	2.8 [2.7, 2.8]	0.49
	Probability [95% CI]						
	*n* = 1919	*n* = 2535	*n* = 2732	*n* = 3111	*n* = 2427	n = 2563	p interaction SEP*Time
Fruit (≥ 2 serves/d)	0.46 [0.43, 0.49]	0.48 [0.45, 0.51]	0.52 [0.50, 0.55]	0.45 [0.43, 0.48]***	0.54 [0.51, 0.56]	0.49 [0.47, 0.52]*	**0.002**
Vegetables (≥ 4 serves/d)	0.17 [0.15, 0.19]	0.19 [0.17, 0.21]	0.20 [0.18, 0.22]	0.18 [0.17, 0.20]	0.20 [0.18, 0.22]	0.18 [0.16, 0.20]	0.16
	MEN, mean [95% CI]						
	Low income		Medium income		High income		
	1995 *n* = 1115	2011–13 *n* = 1014	1995 *n* = 1731	2011–13 *n* = 1484	1995 *n* = 1652	2011–13 *n* = 1399	p interaction SEP*Time
Energy (kJ)	10,690 [10,390, 10,990]	9874 [9561, 10,186]	11,263 [11,028, 11,499]	10,014 [9764, 10,264]	11,350 [11,111, 11,589]	10,316 [10,053, 10,579]	0.28
Carbohydrate (%E)	44.9 [44.2, 45.6]	44.7 [43.8, 45.6]	44.7 [44.2, 45.3]	44.0 [43.4, 44.7]	43.7 [43.2, 44.3]	41.3 [40.5, 42.1]***	**0.003**
Total sugars (%E)	19.0 [18.4, 19.7]	18.8 [18.0, 19.5]	19.3 [18.8, 19.8]	19.0 [18.4, 19.6]	18.9 [18.4, 19.5]	18.0 [17.4, 18.6]	0.45
Protein (%E)	16.5 [16.1, 16.9]	17.5 [17.0, 18.0]	16.7 [16.4, 16.9]	18.0 [17.7, 18.4]	16.9 [16.6, 17.2]	18.7 [18.2, 19.3]	0.12
Fat (%E)	31.5 [31.0, 32.1]	31.0 [30.3, 31.7]	32.2 [31.7, 32.6]	30.2 [29.7, 30.8]***	31.4 [31.0, 31.9]	30.6 [29.9, 31.2]*	**0.028**
SFA (%E)	12.2 [11.9, 12.5]	12.2 [11.8, 12.5]	12.8 [12.5, 13.0]	11.3 [11.1, 11.6]***	12.3 [12.1, 12.6]	11.3 [10.9, 11.6]***	**< 0.0001**
MUFA (%E)	11.7 [11.4, 11.9]	11.7 [11.4, 12.1]	11.7 [11.5, 11.9]	11.6 [11.3, 11.9]	11.6 [11.4, 11.8]	11.8 [11.6, 12.1]	0.29
PUFA (%E)	4.8 [4.6, 5.0]	4.4 [4.2, 4.6]**	4.8 [4.7, 5.0]	4.7 [4.5, 4.8]	4.7 [4.6, 4.8]	4.8 [4.6, 5.0]	**0.017**
Fiber density (g/1000 kJ)	2.0 [1.9, 2.0]	2.6 [2.5, 2.7]	1.9 [1.9, 2.0]	2.6 [2.5, 2.7]	2.0 [1.9, 2.0]	2.6 [2.5, 2.6]	0.26
	Probability [95% CI]						
	*n* = 803	*n* = 1015	*n* = 1315	n = 1484	*n* = 1228	n = 1399	p interaction SEP*Time
Fruit (≥ 2 serves/d)	0.41 [0.37, 0.45]	0.44 [0.39, 0.48]	0.49 [0.46, 0.52]	0.39 [0.36, 0.42]***	0.49 [0.46, 0.52]	0.43 [0.39, 0.46]**	**0.003**
Vegetables (≥ 4 serves/d)	0.14 [0.11, 0.17]	0.18 [0.15, 0.22]	0.18 [0.15, 0.20]	0.16 [0.14, 0.19]	0.15 [0.13, 0.18]	0.14 [0.12, 0.17]	0.07
	WOMEN, mean [95% CI]						
	Low income		Medium income		High income		
	1995 *n* = 1492	2011–13 *n* = 1520	1995 *n* = 1793	2011–13 *n* = 1626	1995 *n* = 1494	2011–13 *n* = 1164	p interaction SEP*Time
Energy (kJ)	7229 [7058, 7400]	7121 [6917, 7325]	7436 [7274, 7598]	7466 [7286, 7647]	7950 [7775, 8126]	7675 [7434, 7917]	0.29
Carbohydrate (%E)	47.1 [46.5, 47.8]	45.7 [44.9, 46.5]**	46.3 [45.8, 46.8]	43.5 [42.7, 44.2]***	44.7 [44.2, 45.3]	41.2 [40.3, 42.1]***	**0.017**
Total sugars (%E)	20.7 [20.1, 21.3]	20.9 [20.2, 21.6]	20.6 [20.1, 21.1]	19.6 [19.0, 20.2]	19.8 [19.3, 20.3]	18.9 [18.3, 19.6]	0.13
Protein (%E)	16.7 [16.4, 17.0]	18.3 [17.8, 18.7]	16.8 [16.5, 17.1]	18.5 [18.1, 18.9]	17.0 [16.7, 17.3]	18.8 [18.3, 19.3]	0.86
Fat (%E)	31.5 [30.9, 32.1]	30.6 [29.9, 31.3]	31.7 [31.2, 32.1]	31.3 [30.7, 31.9]	31.9 [31.4, 32.5]	31.7 [31.0, 32.4]	0.51
SFA (%E)	12.1 [11.8, 12.4]	11.6 [11.2, 11.9]	12.3 [12.1, 12.6]	11.6 [11.3, 11.9]	12.5 [12.2, 12.8]	11.4 [11.1, 11.8]	0.23
MUFA (%E)	11.3 [11.1, 11.6]	11.6 [11.2, 11.9]	11.4 [11.2, 11.6]	11.9 [11.6, 12.2]	11.5 [11.2, 11.7]	12.2 [11.8, 12.6]	0.23
PUFA (%E)	5.0 [4.9, 5.2]	4.7 [4.5, 4.9]*	4.9 [4.8, 5.0]	5.0 [4.8, 5.2]	4.9 [4.8, 5.1]	5.3 [5.0, 5.5]*	**0.001**
Fiber density (g/1000 kJ)	2.3 [2.2, 2.4]	3.0 [2.9, 3.1]	2.3 [2.2, 2.3]	2.8 [2.8, 2.9]	2.3 [2.2, 2.4]	3.0 [2.9, 3.1]	0.34
	Probability [95% CI]						
	*n* = 1116	n = 1520	*n* = 1417	*n* = 1627	*n* = 1199	n = 1164	p interaction SEP*Time
Fruit (≥ 2 serves/d)	0.52 [0.48, 0.55]	0.53 [0.49, 0.56]	0.56 [0.53, 0.59]	0.52 [0.49, 0.55]	0.59 [0.55, 0.62]	0.57 [0.53, 0.61]	0.34
Vegetables (≥ 4 serves/d)	0.20 [0.18, 0.23]	0.20 [0.18, 0.23]	0.22 [0.19, 0.25]	0.21 [0.18, 0.23]	0.24 [0.21, 0.27]	0.23 [0.20, 0.26]	0.84

^a^*Abbreviations: %E* % of energy, *SEP* socioeconomic position

Asterisks indicate significantly different compared to 1995 value *p < 0.05, **p < 0.01, ***p < 0.001

Values represent survey-weighted, covariate adjusted means (or probabilities where indicated) and 95% CI

Linear and logistic regression models were adjusted for age, sex/gender (except in stratified analyses), and smoking. Where results of the overall F test were statistically significant, pairwise comparisons were performed to examine change in intakes over time according to household income

Income is expressed as deciles of gross equivalized household income. Low income: deciles 1–3; Medium income: deciles 4–7; High income: deciles 8–10

In stratified analyses, trends in consumption of carbohydrate, fat, SFA, PUFA and fruit varied over time by income level among men, whereas among women only carbohydrate and PUFA intakes changed over time (Table 3).

#### Area-level disadvantage

Trends in consumption of carbohydrate, total fat, MUFA, and PUFA varied over time by area-level disadvantage (Table 4). Carbohydrate intake declined in a graded manner by 1.1%E − 1.3%E in the three most disadvantaged quintiles (*p* < 0.05), and by 2.6%E and 3.3%E in the two least disadvantage quintiles (p < 0.001). Fat intake declined by 1.1%E − 1.4%E in the three most disadvantaged quintiles (*p* < 0.01), whereas it was unchanged in the two least disadvantaged quintiles. MUFA intake was unchanged in the three most disadvantaged quintiles, but increased by 0.7%E (p < 0.001) and by 0.5%E (*p* = 0.002) in the two least disadvantaged quintiles. PUFA intake declined by 0.4%E (*p* = 0.007) in the most disadvantaged quintile and by 0.2%E in the third most disadvantaged quintile (*p* = 0.03), with no changes in the others. There were no changes over time according to area-level disadvantage in dietary intakes of energy, total sugars, protein, SFA, fiber, fruit or vegetables.

**Table 4:** Dietary intake according to area-level disadvantage in Australian adults in the 1995 National Nutrition Survey and the 2011–13 National Nutrition and Physical Activity Survey^a^

	OVERALL, mean [95% CI]										
	Quintile 1 Most disadvantaged		Quintile 2		Quintile 3		Quintile 4		Quintile 5 Least disadvantaged		
	1995 *n* = 1778	2011–13 *n* = 1582	1995 *n* = 1787	2011–13 *n* = 1719	1995 *n* = 1810	2011–13 *n* = 1633	1995 *n* = 1953	2011–13 *n* = 1453	1995 *n* = 1949	2011–13 *n* = 1820	p interaction SEP*Time
Energy (kJ)	9073 [8859, 9287]	8628 [8393, 8864]	9383 [9164, 9602]	8607 [8384, 8829]	9482 [9260, 9704]	8892 [8683, 9100]	9405 [9201, 9610]	8813 [8571, 9054]	9562 [9356, 9768]	8945 [8738, 9151]	0.69
Carbohydrate (%E)	45.6 [45.0, 46.1]	44.5 [43.8, 45.2]*	45.4 [44.8, 46.0]	44.1 [43.4, 44.8]**	44.8 [44.3, 45.4]	43.7 [43.0, 44.5]*	45.3 [44.8, 45.8]	42.7 [42.0, 43.4]***	45.1 [44.5, 45.6]	41.8 [41.2, 42.5]***	**0.001**
Total sugars (%E)	20.3 [19.7, 20.9]	19.6 [18.9, 20.2]	19.9 [19.4, 20.4]	19.3 [18.7, 19.9]	19.6 [19.1, 20.1]	19.7 [19.1, 20.2]	19.6 [19.1, 20.1]	19.3 [18.7, 19.9]	19.4 [18.9, 19.9]	18.1 [17.6, 18.6]	0.17
Protein (%E)	16.9 [16.6, 17.2]	18.0 [17.6, 18.4]	16.7 [16.4, 16.9]	18.3 [17.8, 18.7]	16.8 [16.5, 17.0]	18.3 [17.9, 18.7]	16.8 [16.6, 17.1]	18.6 [18.2, 19.0]	16.8 [16.5, 17.0]	18.4 [18.1, 18.8]	0.39
Fat (%E)	31.9 [31.4, 32.4]	30.8 [30.2, 31.4]**	31.9 [31.4, 32.5]	30.5 [29.9, 31.1]***	32.1 [31.6, 32.6]	30.7 [30.2, 31.3]***	31.3 [30.9, 31.8]	31.4 [30.7, 32.0]	31.4 [31.0, 31.9]	31.0 [30.4, 31.5]	**0.026**
SFA (%E)	12.4 [12.2, 12.7]	11.7 [11.3, 12.0]	12.5 [12.2, 12.8]	11.4 [11.2, 11.7]	12.5 [12.3, 12.8]	11.6 [11.4, 11.9]	12.3 [12.0, 12.5]	11.6 [11.3, 11.9]	12.3 [12.1, 12.6]	11.3 [11.1, 11.6]	0.72
MUFA (%E)	11.6 [11.4, 11.8]	11.8 [11.5, 12.1]	11.7 [11.4, 11.9]	11.6 [11.3, 11.9]	11.7 [11.5, 11.9]	11.7 [11.5, 12.0]	11.3 [11.1, 11.5]	12.0 [11.7, 12.3]***	11.4 [11.2, 11.6]	11.9 [11.6, 12.2]**	**0.010**
PUFA (%E)	5.0 [4.8, 5.1]	4.6 [4.5, 4.8]**	4.8 [4.7, 5.0]	4.7 [4.6, 4.9]	4.9 [4.8, 5.1]	4.7 [4.5, 4.8]*	4.8 [4.7, 4.9]	5.0 [4.8, 5.2]	4.8 [4.7, 4.9]	5.0 [4.8, 5.1]	**0.002**
Fiber density (g/1000 kJ)	2.1 [2.1, 2.2]	2.7 [2.6, 2.8]	2.1 [2.0, 2.1]	2.8 [2.7, 2.9]	2.1 [2.1, 2.2]	2.7 [2.6, 2.8]	2.2 [2.1, 2.2]	2.8 [2.7, 2.9]	2.1 [2.1, 2.2]	2.8 [2.7, 2.9]	0.54
	Probability [95% CI]										
	*n* = 1325	n = 1582	*n* = 1355	*n* = 1721	*n* = 1378	n = 1633	*n* = 1510	n = 1453	n = 1510	n = 1820	p interaction SEP*Time
Fruit (≥ 2 serves/d)	0.48 [0.44, 0.51]	0.46 [0.43, 0.50]	0.54 [0.50, 0.57]	0.47 [0.44, 0.50]	0.51 [0.47, 0.54]	0.48 [0.45, 0.51]	0.52 [0.49, 0.55]	0.49 [0.46, 0.52]	0.51 [0.48, 0.55]	0.47 [0.44, 0.50]	0.60
Vegetables (≥ 4 serves/d)	0.18 [0.15, 0.20]	0.19 [0.17, 0.22]	0.18 [0.16, 0.21]	0.19 [0.17, 0.22]	0.21 [0.18, 0.25]	0.20 [0.18, 0.23]	0.20 [0.17, 0.23]	0.19 [0.16, 0.21]	0.18 [0.15, 0.20]	0.16 [0.14, 0.18]	0.59
	MEN, mean [95% CI]										
	Quintile 1 Most disadvantaged		Quintile 2		Quintile 3		Quintile 4		Quintile 5 Least disadvantaged		
	1995 *n* = 829	2011–13 *n* = 717	1995 *n* = 863	2011–13 *n* = 819	1995 *n* = 881	2011–13 *n* = 779	1995 *n* = 943	2011–13 *n* = 719	1995 *n* = 982	2011–13 n = 863	p interaction SEP*Time
Energy (kJ)	10,751 [10,416, 11,086]	10,067 [9680, 10,454]	11,142 [10,790, 11,493]	9803 [9452, 10,155]	11,380 [11,042, 11,718]	10,148 [9825, 10,470]	11,152 [10,843, 11,461]	10,322 [9963, 10,682]	11,275 [10,961, 11,589]	10,101 [9782, 10,420]	0.28
Carbohydrate (%E)	44.6 [43.8, 45.4]	43.9 [42.9, 44.9]	44.5 [43.8, 45.3]	44.1 [43.1, 45.0]	43.7 [42.9, 44.4]	43.6 [42.6, 44.5]	44.7 [44.0, 45.5]	42.5 [41.5, 43.5]***	44.4 [43.7, 45.2]	42.2 [41.3, 43.1]***	**0.034**
Total sugars (%E)	19.7 [19.0, 20.5]	18.3 [17.4, 19.2]	19.1 [18.4, 19.8]	19.1 [18.2, 20.1]	18.9 [18.2, 19.7]	18.8 [18.0, 19.6]	19.1 [18.4, 19.8]	18.5 [17.7, 19.4]	18.7 [18.0, 19.5]	18.1 [17.4, 18.8]	0.44
Protein (%E)	16.8 [16.3, 17.3]	17.8 [17.2, 18.4]	16.8 [16.4, 17.1]	18.1 [17.4, 18.8]	16.8 [16.4, 17.2]	18.4 [17.8, 18.9]	16.6 [16.2, 16.9]	18.5 [17.9, 19.0]	16.7 [16.3, 17.0]	18.0 [17.5, 18.5]	0.47
Fat (%E)	32.2 [31.6, 32.8]	30.6 [29.7, 31.4]	31.9 [31.3, 32.5]	30.0 [29.2, 30.9]	32.1 [31.4, 32.7	30.6 [29.9, 31.3]	31.5 [30.9, 32.1]	31.2 [30.4, 32.0]	31.3 [30.7, 31.9]	30.3 [29.6, 31.1]	0.22
SFA (%E)	12.6 [12.3, 13.0]	11.6 [11.1, 12.0]	12.4 [12.1, 12.8]	11.3 [10.9, 11.7]	12.5 [12.2, 12.9]	11.6 [11.2, 12.0]	12.5 [12.2, 12.9]	11.7 [11.3, 12.2]	12.3 [12.0, 12.6]	11.4 [11.0, 11.8]	0.94
MUFA (%E)	11.8 [11.5, 12.1]	11.9 [11.4, 12.3]	11.8 [11.5, 12.1]	11.5 [11.1, 11.9]	11.8 [11.5, 12.1]	11.7 [11.3, 12.0]	11.4 [11.2, 11.7]	12.0 [11.6, 12.4]	11.5 [11.2, 11.7]	11.6 [11.3, 12.0]	0.13
PUFA (%E)	4.8 [4.6, 5.0]	4.5 [4.3. 4.7]	4.8 [4.6, 5.0]	4.6 [4.4, 4.8]	4.9 [4.7, 5.1]	4.7 [4.5, 4.9]	4.7 [4.5, 4.9]	4.8 [4.6, 5.1]	4.7 [4.5, 4.8]	4.7 [4.4, 4.9]	0.26
Fiber density (g/1000 kJ)	2.0 [1.9, 2.0]	2.6 [2.4, 2.7]	1.9 [1.9, 2.0]	2.6 [2.5, 2.7]	2.0 [1.9, 2.0]	2.6 [2.5, 2.7]	2.0 [1.9, 2.0]	2.6 [2.5, 2.7]	2.0 [1.9, 2.0]	2.6 [2.5, 2.7]	0.99
	Probability [95% CI]										
	*n* = 611	n = 717	*n* = 635	n = 820	*n* = 656	n = 779	*n* = 706	n = 719	*n* = 738	n = 863	p interaction SEP*Time
Fruit (≥ 2 serves/d)	0.42 [0.38, 0.47]	0.40 [0.35, 0.45]	0.51 [0.46, 0.55]	0.40 [0.36, 0.44]	0.48 [0.43, 0.52]	0.42 [0.37, 0.46]	0.48 [0.44, 0.53]	0.46 [0.41, 0.50]	0.46 [0.42, 0.51]	0.40 [0.35, 0.44]	0.39
Vegetables (≥ 4 serves/d)	0.13 [0.10, 0.16]	0.17 [0.14, 0.21]	0.17 [0.14, 0.21]	0.18 [0.14, 0.22]	0.18 [0.15, 0.22]	0.16 [0.13, 0.20]	0.17 [0.14, 0.21]	0.17 [0.13, 0.20]	0.14 [0.11, 0.17]	0.12 [0.09, 0.15]	0.27
	WOMEN, mean [95% CI]										
	Quintile 1 Most disadvantaged		Quintile 2		Quintile 3		Quintile 4		Quintile 5 Least disadvantaged		
	1995 *n* = 949	2011–13 *n* = 865	1995 *n* = 924	2011–13 *n* = 900	1995 *n* = 929	2011–13 *n* = 854	1995 *n* = 1010	2011–13 *n* = 734	1995 *n* = 967	2011–13 *n* = 957	p interaction SEP*Time
Energy (kJ)	7307 [7074, 7540]	7130 [6874, 7385]	7533 [7319, 7748]	7338 [7092, 7585]	7461 [7220, 7703]	7592 [7341, 7843]	7551 [7340, 7762]	7230 [6929, 7531]	7740 [7538, 7943]	7741 [7482, 8001]	0.41
Carbohydrate (%E)	46.5 [45.7, 47.3]	45.2 [44.2, 46.2]*	46.3 [45.5, 47.1]	44.2 [43.2, 45.1]**	46.1 [45.4, 46.7]	44.0 [42.9, 45.0]**	45.9 [45.3, 46.6]	42.9 [41.8, 44.0]***	45.8 [45.1, 46.5]***	41.5 [40.5, 42.5]	**0.008**
Total sugars (%E)	20.8 [20.1, 21.6]	20.9 [20.0, 21.8]	20.7 [20.0, 21.5]	19.4 [18.7, 20.1]**	20.3 [19.7, 21.0]	20.6 [19.7, 21.4]	20.1 [19.5, 20.8]	20.2 [19.3, 21.0]	20.0 [19.4, 20.7]	18.3 [17.5, 19.0]***	**0.016**
Protein (%E)	17.0 [16.6, 17.4]	18.3 [17.7, 18.8]	16.6 [16.2, 16.9]	18.4 [17.9, 18.9]	16.7 [16.3, 17.1]	18.2 [17.7, 18.8]	17.0 [16.7, 17.4]	18.8 [18.3, 19.3]	16.9 [16.5, 17.2]	18.9 [18.3, 19.4]	0.62
Fat (%E)	31.7 [31.0, 32.4]	31.0 [30.1, 31.9]	32.0 [31.3, 32.8]	31.0 [30.2, 31.8]	32.1 [31.5, 32.8]	30.9 [30.1, 31.7]	31.1 [30.5, 31.8]	31.5 [30.6, 32.5]	31.6 [31.0, 32.2]	31.6 [30.8, 32.4]	0.14
SFA (%E)	12.2 [11.8, 12.5]	11.8 [11.3, 12.3]	12.5 [12.1, 12.9]	11.6 [11.2, 12.0]	12.5 [12.2, 12.9]	11.7 [11.2, 12.1]	12.0 [11.7, 12.3]	11.4 [11.0, 11.9]	12.3 [12.0, 12.7]	11.3 [10.9, 11.7]	0.51
MUFA (%E)	11.4 [11.1, 11.7]	11.7 [11.3, 12.1]	11.6 [11.2, 11.9]	11.7 [11.4, 12.1]	11.6 [11.3, 11.9]	11.8 [11.4, 12.1]	11.2 [10.9, 11.5]	12.1 [11.6, 12.5]	11.3 [11.0, 11.5]	12.2 [11.8, 12.6]	0.07
PUFA (%E)	5.1 [4.9, 5.3]	4.8 [4.5, 5.0]	4.9 [4.7, 5.1]	4.9 [4.7, 5.1]	4.9 [4.7, 5.2]	4.7 [4.5, 4.9]	4.9 [4.7, 5.1]	5.3 [4.9, 5.6]*	4.9 [4.7, 5.1]	5.3 [5.0, 5.5]*	**0.004**
Fiber density (g/1000 kJ)	2.3 [2.2, 2.3]	2.9 [2.8, 3.0]	2.2 [2.1, 2.3]	3.0 [2.8, 3.2]	2.3 [2.2, 2.4]	2.9 [2.7, 3.0]	2.4 [2.3, 2.4]	3.0 [2.9, 3.2]	2.3 [2.2, 2.3]	2.9 [2.8, 3.1]	0.31
	Probability [95% CI]										
	*n* = 714	n = 865	*n* = 720	*n* = 901	*n* = 722	n = 854	*n* = 804	n = 734	*n* = 772	n = 957	p interaction SEP*Time
Fruit (≥ 2 serves/d)	0.53 [0.49, 0.57]	0.53 [0.48, 0.57]	0.57 [0.52, 0.61]	0.55 [0.51, 0.59]	0.54 [0.49, 0.58]	0.54 [0.49, 0.58]	0.56 [0.52, 0.60]	0.52 [0.48, 0.57]	0.57 [0.53, 0.61]	0.54 [0.50, 0.58]	0.90
Vegetables (≥ 4 serves/d)	0.23 [0.19, 0.26]	0.22 [0.18, 0.26]	0.20 [0.16, 0.23]	0.20 [0.17, 0.24]	0.24 [0.20, 0.29]	0.24 [0.20, 0.28]	0.23 [0.19, 0.26]	0.21 [0.17, 0.25]	0.21 [0.18, 0.25]	0.19 [0.16, 0.23]	0.95

^a^*Abbreviations: %E* % of energy, *SEP* socioeconomic position

Asterisks indicate significantly different compared to 1995 value *p < 0.05, **p ≤ 0.01, ***p < 0.001

Macronutrients are reported as % of total energy (%E)

Values represent survey-weighted, covariate adjusted means (or probabilities where indicated) and 95% CI

Linear and logistic regression models were adjusted for age, sex/gender (except in stratified analyses), and smoking. Where results of the overall F test were statistically significant, pairwise comparisons were performed to examine change in intakes over time according to educational level

The Socio-Economic Index for Areas Index of Relative Socio-Economic Disadvantage was used to define area-level disadvantage. The index is divided into quintiles ordered from most (quintile 1) to least disadvantaged (quintile 5). Higher scores are indicative of an area with lower levels of disadvantage, such as where fewer individuals have low incomes, low educational attainment, or work in unskilled occupations

Bold data indicates statistically significant differences (*p*  < 0.05)

In stratified analyses, only carbohydrate intake varied over time by area-level disadvantage among men (Table 4). Among women, trends in consumption of carbohydrate, total sugars, and PUFA varied over time by area-level disadvantage.

#### Summary of overall trends

**Fig. 1:**
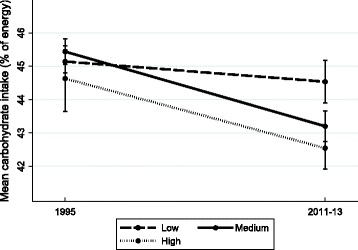
Trends in mean proportion of energy from carbohydrate according to level of education in Australian adults, 1995 to 2011–13. Values represent survey-weighted, covariate adjusted means and 95% CI estimated using the margins command in Stata. Time x education interaction *p* = 0.0014

**Fig. 2:**
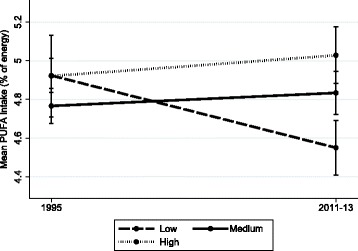
Trends in mean proportion of energy from polyunsaturated fat according to level of education in Australian adults, 1995 to 2011–13. Values represent survey-weighted, covariate adjusted means and 95% CI estimated using the margins command in Stata. Time x education interaction *p* = 0.0001

**Fig. 3:**
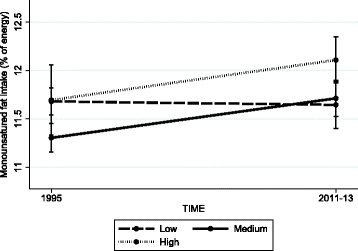
Trends in mean proportion of energy from monounsaturated fat according to level of education in Australian adults, 1995 to 2011–13. Values represent survey-weighted, covariate adjusted means and 95% CI estimated using the margins command in Stata. Time x education interaction *p* = 0.0364

Changes in dietary intake according to SEP were most consistent for carbohydrate, PUFA, and MUFA (Table 5). Between 1995 and 2011–13 carbohydrate intake declined in a graded manner according to all three SEP indicators, with greater declines in higher relative to lower SEP groups. PUFA intakes declined in the lowest SEP groups according to all three measures, and remained stable or increased in higher SEP groups. MUFA intakes were stable in those with a low educational level and in those living in more disadvantaged areas, but increased in those with a medium educational level (with a trend in the high education group; *p* = 0.06) and in those living in less disadvantaged areas. Thus for all three nutrients, more favorable changes were observed in higher SEP groups, and dietary inequalities widened (Figs. 1, 2 and 3 depict changes according to educational level). Intake of energy, total fat, SFA and fruit differed over time according to a single SEP measure (Table 5). There were no changes in intake of total sugars, protein, fiber or vegetables according to any SEP measures.

**Table 5:** Change in dietary intake between 1995 and 2011–13 according to socioeconomic position in Australian adults

	OVERALL										
	Energy	Carbohydrate	Total sugars	Protein	Total fat	SFA	MUFA	PUFA	Fiber	Fruit	Vegetables
Educational level	**√**	**√**					**√**	**√**			
Household income		**√**				**√**		**√**		**√**	
Area-level disadvantage		**√**			**√**		**√**	**√**			
MEN											
Educational level											
Household income		**√**			**√**	**√**		**√**		**√**	
Area-level disadvantage		**√**									
WOMEN											
Educational level		**√**					**√**	**√**			
Household income		**√**						**√**			
Area-level disadvantage		**√**	**√**					**√**			

√ indicates significant change in intake in linear and logistic regression models adjusted for age, sex/gender (except in stratified analyses), and smoking between 1995 and 2011–13 according to socioeconomic position (within one or more categories)

In stratified analyses among women, consistent evidence of change in intakes of carbohydrate and PUFA was observed according to all three SEP measures, whereas total sugars and MUFA differed over time according to a single measure of SEP. Among men, carbohydrate differed according to two, and total fat, SFA, PUFA and fruit differed according to one SEP measure over time. Overall, widening inequalities in intakes of carbohydrate, PUFA and MUFA were predominantly driven by changes in women’s intakes, with smaller changes in men observed.

## Discussion

There were few changes in the dietary intakes of Australian adults between 1995 and 2011–13 according to three measures of SEP, with the exceptions of carbohydrate, PUFA, and MUFA. For these three nutrients, small, but more favorable changes in intakes were observed in higher SEP groups, particularly in women. Limited evidence of widening dietary inequalities, especially in dietary factors that often signal high intakes of discretionary foods (e.g. SFA, total sugars), is a positive finding from a public health perspective. Nevertheless, dietary intakes remained poor across the SEP spectrum, as evidenced by high consumption of SFA and total sugars, and low fiber, fruit and vegetable intakes. That intakes remained poor across the socioeconomic spectrum accords with similar evidence from the US [15–17], and points to the reality that the drivers of unhealthy diets are pervasive and influence all of society, regardless of SEP.

Evidence from several studies indicates that energy and macronutrient intakes have remained relatively stable in the US according to SEP since 1971 [15, 38]. Similar results were found in the Netherlands between 1987/88 and 1997/98 [41], and in Canada from 1986 to 2001 (according to purchasing data) [42]. This relative stability in energy and macronutrient intakes across several nations, over multiple decades, and using a variety of SEP measures is consistent with our findings. Nevertheless, between 1999 and 2012, richer and more educated subgroups in the US experienced greater improvements in diet quality and in intakes of some foods (e.g. whole fruit, refined grains) [15]. It is possible that dietary differentials are more difficult to discern at the macronutrient level.

Carbohydrate intake declined within all SEP groups over time in a graded manner, with greater declines in more advantaged groups. Given that intakes of fiber, total sugars, fruits and vegetables and other macronutrients demonstrated little to no concurrent change, it is plausible that these changes were due to reduced intake of refined grains. This supposition accords with evidence that lower SEP groups tend to consume more refined and fewer whole grains [27], and that intake of refined grains declined among higher SEP groups between 1999 and 2012 in the US [15]. It appears as though small declines in carbohydrate were partially counterbalanced by small increases in PUFA and MUFA in higher SEP groups. By contrast, low SEP groups reduced their intake of PUFA with no change in MUFA intakes, in opposition to dietary guidelines that recommend replacing SFA with PUFA and MUFA [43].

In the current study, the probability of meeting fruit intake recommendations only differed over time according to household income, with no changes in differentials in vegetable intakes. Notably, however, fruit and vegetable intake remained far from optimal. Disparities in fruit and vegetable intakes have been relatively stable on an international level for several decades, including in the US from 1965 to 2012 [15, 44], in France from 1985/87 to 1995/97 [45], in the Netherlands from 1987/88 to 1997/98 [41], in Scotland from 1986 to 1995 [46], and in Canada from 1986 to 2001 [42].

Income, education and area-level disadvantage reflect distinct, yet overlapping axes of stratification, as illustrated by our findings [47]. Multiple mechanisms may explain distinct linkages between various measures of SEP and dietary behaviors. For instance, more highly educated individuals tend to have greater nutrition-related knowledge [48–50], and thus may be more aware of dietary recommendations. Education is also a strong determinant of future employment and income [51], which may in turn influence access to social and economic resources in support of healthy eating. Income is the indicator of SEP that most directly captures access to material resources [47], which may influence access to healthful foods [52, 53]. Finally, area-level measures can be theorized as measures of contextual effects, and have been shown to have independent effects on diet [54]. A comprehensive understanding of the mechanisms underlying our findings requires a fuller description of the types of foods from which these dietary components were derived, in addition to contextual information about the various individual, social and environmental factors that drove their intake.

Sex/gender differences in dietary intakes intersected with SEP, such that increasing inequalities in intakes of carbohydrate, PUFA and MUFA were predominantly driven by changes in women. In addition, women were susceptible to all three forms of disadvantage, whereas for men, low household income was the primary driver of inequalities. The reasons for these differences are unclear, however familial factors might be implicated. Women are more likely to head single parent households than men [55], which might make them more susceptible to disadvantage if they compromise their own intake to shield their children from hunger [56, 57]. Furthermore, women are still primarily responsible for food selection and preparation [58], their educational level rather than men’s might be more predictive of their own and their partners’ intakes.

### Implications

Australia is commonly portrayed as a more progressive liberal welfare state compared to many other developed nations, including the US [59, 60]. Nevertheless, despite major social policy reforms undertaken between 2007 and 2013 which aimed to reduce economic, social and health inequalities [61], income inequality has been trending upward in Australia over the last two decades, albeit at a slower rate than in the US [60, 62]. It is not clear to what degree and over what time frame dietary inequalities may be sensitive to changes in socioeconomic inequalities, particularly given that dietary intakes remain poor across the socioeconomic spectrum [33]. However, evidence presented here suggests that inequalities in some aspects of dietary intake may be responsive to broader long-term socioeconomic trends such as these. Means-testing and residual services are common in Australia [61, 63]. It is possible that universal approaches that directly tackle structural determinants of health might offer a more effective policy response to inequalities [61].

### Strengths and limitations

This study provides the first ever nationally representative estimates of change in dietary intake according to both individual and area-level measures of SEP over a 16 year period in Australian adults. We adopted a gradient approach, examining change in dietary intake across the SEP spectrum, and used multiple measures of SEP that tap into distinct mechanisms through which inequalities are generated and maintained.

Methodologic changes between surveys may have influenced our results to a small degree. First, dietary recalls were conducted using a three-stage multiple pass method in 1995, and a five-stage method in 2011–13, and the latter may have helped to minimize omitted and forgotten foods. Second, similar to previous analyses [15, 16], data are not adjusted for residence in an urban or rural location, or for country of birth, due to differences in which these data were coded between surveys. Thus, there may be residual confounding. Third, questions on frequency of fruit and vegetable intake were modified slightly in 2011–13 to specifically request that participants include potatoes and exclude juices from their estimates, whereas treatment of these foods was not specified in 1995. Because the impact of these methodological changes was not investigated, time and method effects may be confounded in some cases, however the relative stability of dietary intake according to SEP in most cases suggests these methodologic changes were not a major concern.

Although the degree of energy intake underreporting by SEP did not change across survey years, underreporting is known to have increased over time, particularly among men [20]. Therefore, while our estimates of changes in dietary intake according to SEP remain robust, caution is advised in attempts to assess the presence or strength of socioeconomic gradients *within* a particular survey cycle, as differential misreporting according to SEP may have biased these estimates [64–67].

Methodologic differences in the manner in which mixed dishes were disaggregated in the two surveys precluded investigation of change in dietary inequalities at all levels of the nutritional hierarchy (e.g. nutrients, foods, eating occasions, dietary patterns) [20]. However our results may still provide an indication of change at higher levels, as healthful dietary patterns are identified on the basis of their relative content of constituent foods, which are themselves defined on the basis of the quality and quantity of the nutrients they contain [18]. Moreover, fruit and vegetable intake correlates strongly with overall diet quality [68]. Understanding trajectories of change in nutrient intakes remains important because nutrients represent the level that most closely links dietary intake with the physiology of health and disease [69]. Thus, our analyses can enhance mechanistic understanding of the contribution of dietary inequalities to inequalities in obesity and chronic disease.

## Conclusions

We found consistent evidence of more favorable changes in dietary intakes of carbohydrate, PUFA and MUFA in higher SEP groups between 1995 and 2011–13 compared to lower SEP groups. There were few changes in dietary intakes of energy, other macronutrients, fiber, fruits and vegetables. Despite the persistence of suboptimal dietary intakes, limited evidence of widening dietary inequalities is a positive finding from a public health perspective. Nevertheless, as income inequality has been trending upward in Australia, it will be important to examine change in inequalities at the level of dietary patterns as these data become available.

## Additional file

Additional file 1: 1995 National Nutrition Survey flow diagram. (PDF 10 kb)

Additional file 2: 2011–13 National Nutrition and Physical Activity Survey flow diagram. (PDF 94 kb)

## Abbreviations

%E: % energy
MUFA: Monounsaturated fat
NNPAS: National Nutrition and Physical Activity Survey
NNS: National Nutrition Survey
PUFA: Polyunsaturated fat
SEIFA: Socio-Economic Index for Areas
SEP: Socioeconomic position
SFA: Saturated fat
