# Supplementary figures and images for: Are dietary inequalities among Australian adults changing? a nationally representative analysis of dietary change according to socioeconomic position between 1995 and 2011–13
Source: Int J Behav Nutr Phys Act. 2018 Apr 2;15:30. doi: 10.1186/s12966-018-0666-4 (PMC5879763; doi:10.1186/s12966-018-0666-4)

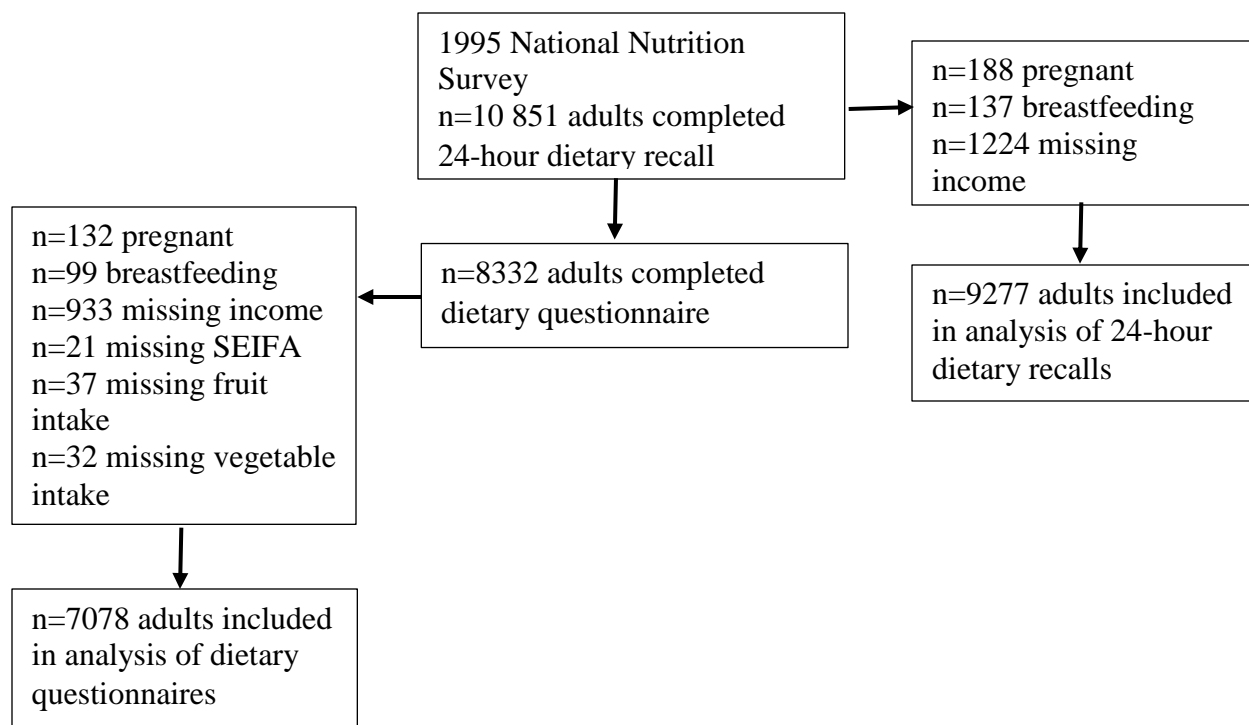

**Additional File 1.** 1995 National Nutrition Survey flow diagram

Supplement: Supplementary file 1 — 1995 National Nutrition Survey flow diagram. (PDF 10 kb) [file 12966_2018_666_MOESM1_ESM.pdf]

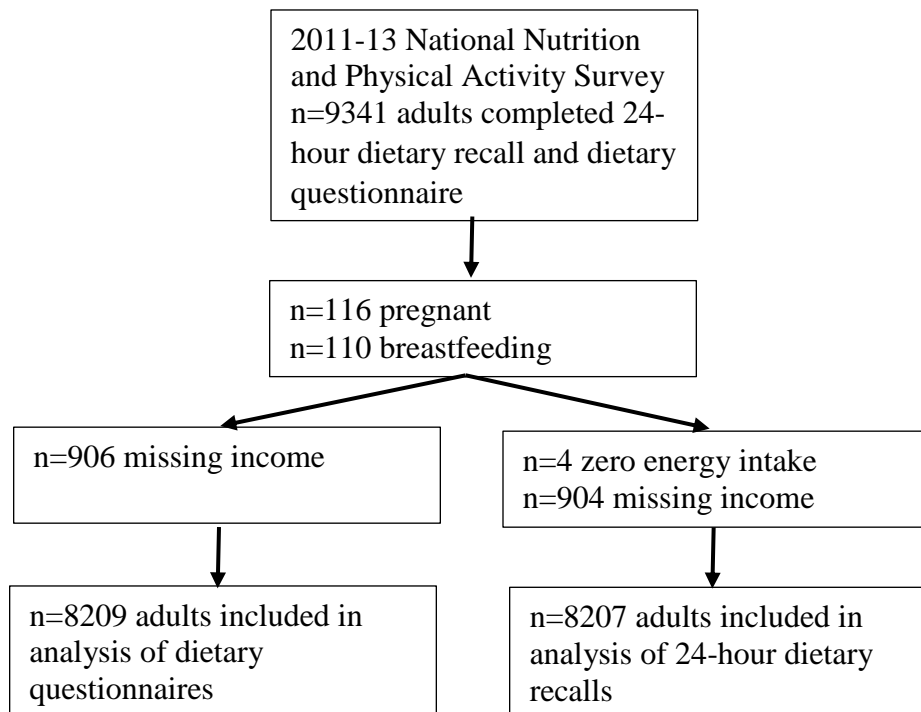

**Additional File 2.** 2011-13 National Nutrition and Physical Activity Survey flow diagram

Supplement: Supplementary file 2 — 2011–13 National Nutrition and Physical Activity Survey flow diagram. (PDF 94 kb) [file 12966_2018_666_MOESM2_ESM.pdf]
